# Putamen Stiffness Declines with Age and Is Associated with Implicit Sequence Learning Outcomes

**DOI:** 10.3390/brainsci15090947

**Published:** 2025-08-29

**Authors:** Hyeon Jung Heselton, Aaron T. Anderson, Curtis L. Johnson, Neal J. Cohen, Bradley P. Sutton, Hillary Schwarb

**Affiliations:** 1 Center for Brain, Biology and Behavior, University of Nebraska-Lincoln, Lincoln, NE 68588, USA; jkim61@unl.edu; 2 Beckman Institute for Advanced Science and Technology, University of Illinois at Urbana Champaign, Urbana, IL 61801, USA; aandrsn3@illinois.edu (A.T.A.); bsutton@illinois.edu (B.P.S.); 3 Department of Biomedical Engineering, University of Delaware, Newark, DE 19713, USA; clj@udel.edu; 4 Department of Psychology, University of Illinois at Urbana-Champaign, Champaign, IL 61820, USA; njc@illinois.edu; 5 Carle Illinois College of Medicine, University of Illinois at Urbana-Champaign, Urbana, IL 61801, USA; 6 The Grainger College of Engineering, Bioengineering Department, University of Illinois at Urbana-Champaign, Urbana, IL 61801, USA; 7 Department of Psychology, University of Nebraska-Lincoln, Lincoln, NE 68588, USA

**Keywords:** sequence learning, implicit learning, serial reaction time task, putamen, volume, magnetic resonance elastography, stiffness

## Abstract

Background/Objectives: Sequence learning, the ability to pick up on regularities in our environment to facilitate behavior, is critically dependent on striatal structures in the brain, with the putamen emerging as a critical hub for implicit sequence learning. As the putamen is known to shrink with age, and age-related declines in sequence learning abilities are common, it has been hypothesized that the structural integrity of the putamen is likely related to sequence learning outcomes. However, the structural literature is sparse. One reason may be that traditional structural imaging measures, like volume, are not sufficiently sensitive to measure changes that are related to performance outcomes. We propose that magnetic resonance elastography (MRE), an emerging neuroimaging tool that provides quantitative measures of microstructural integrity, may fill this gap. Methods: In this study, both sequence learning abilities and the structural integrity of the putamen were assessed in 61 cognitively healthy middle-aged and older adults (range: 45–78 years old). Sequence learning was measured via performance on the Serial Reaction Time Task. Putamen integrity was assessed in two ways: first, via standard structural volume assessments, and second, via MRE measures of tissue integrity. Results: Age significantly correlated with both putamen volume and stiffness but not sequence learning scores. While sequence learning scores did not correlate with volume, MRE-derived measures of putamen stiffness were significantly correlated with learning outcomes such that individuals with stiffer putamen showed higher learning scores. A series of control analyses were performed to highlight the specificity and sensitivity of this putamen stiffness–sequence learning relationship. Conclusions: Together these data indicate that microstructural changes that occur in the putamen as we age may contribute to changes in sequence learning outcomes.

## 1. Introduction

As a fundamental process for interacting with the world, sequence learning generally—and implicit sequence learning specifically—has been studied in the lab for several decades. Healthy individuals across the lifespan demonstrate the ability to learn sequences (e.g., [1,2]), though the size of the learning effect is often smaller for older adults (e.g., [3]). Sequence learning is primarily supported by basal ganglia structures in the brain [4] and is disrupted in many neurological conditions that impact the basal ganglia (e.g., in Parkinson’s disease [5,6,7] or Huntington’s disease [8,9]). Basal ganglia volume, specifically the striatal structures, decreases with age (e.g., [10]), and yet there are few reports of how striatum volume supports sequence learning. One possible reason may be limitations related to standard structural imaging measures. In the current study, we apply an emerging structural imaging technique, magnetic resonance elastography (MRE). MRE is capable of quantifying brain tissue mechanical properties to investigate the relationship between brain microstructural integrity and sequence learning abilities. This study focuses on the structural health of the striatum generally, and the putamen in particular.

While multiple paradigms have been used to investigate the cognitive and neural processes engaged when learning implicit motor sequences, the Serial Reaction Time Task (SRTT; first introduced by Nissen and Bullemer [11]) has dominated the field of implicit sequence learning. The SRTT requires participants to respond (typically with button pushes) to stimuli (visual) that follow a repeating pattern or sequence (for reviews see [12,13]). Participants are not informed about the underlying sequence and are instructed to respond to the stimuli as quickly as possible. Across several sequenced blocks, reaction times (RTs) improve, and they do so at a steeper rate compared to unsequenced blocks indicating that accumulating knowledge about the sequence facilitates speeded responses over and above practice alone (e.g., [11,14,15]). More commonly, blocks with novel sequences are also inserted into the experimental design (e.g., [16,17]). Typically, RTs increase when these novel sequence blocks are introduced and this jump in RT (commonly referred to as the “Transfer Effect”) serves as an efficient and practice-independent measure of sequence learning [13].

Research investigating the relationship between sequence learning and aging has been inconsistent. When compared to young adults, older adults are often unimpaired on simple sequences (e.g., [2,18]), but show impaired (but not obliterated) learning with complex sequences (e.g., [3,19,20]). Additionally, among adults ages 65–80, RT data indicate stable learning across older adulthood [3]. To understand some of this ambiguity, it is important to consider how sequence learning is supported in the brain.

While considerable research has focused on identifying the functional neural correlates of sequence learning (reviews see [12,21,22]), meta-analytic data suggest that after controlling for general visual and motor processes, sequence learning is only associated with basal ganglia activation [4]. Structural imaging investigations of patient populations also consistently identify basal ganglia (specifically striatal) structures as essential for sequence learning. For example, Sefcsik and colleagues [23] reported that a patient with a perinatal lesion in the left putamen showed impaired sequence learning on the SRTT compared to matched comparison participants. Similarly, impaired SRTT performance has been reported among stroke patients with both caudate and putamen lesions [24,25]. Yet, the relationship between striatal structure and sequence learning has not been widely reported for healthy individuals generally, and aging individuals specifically.

And yet much is known about the effects of aging on the structural health of the brain (e.g., [26,27]). It is well established that the volume of striatal structures retracts with age (e.g., [10,28,29]). Regionally specific age-related volume reductions have been associated with cognitive declines in many brain structures (for a review see [30]). For example, in aging populations, hippocampal volume correlates with episodic memory performance (e.g., [31]), prefrontal cortex volume correlates with executive function (e.g., [32,33]), and putamen volume with perceptual-motor skill learning [34]. Thus, it is reasonable to think that the structure of the striatum may also be related to implicit sequence learning outcomes. And yet, to the best of our knowledge, there have been no reports relating striatal volume to implicit sequence learning. One possible explanation is that traditional imaging approaches, like volumetry, may not be sufficiently sensitive to age-related small-scale changes that may be meaningful for sequence learning success or failure. The application of tools sensitive to microstructural tissue changes may be useful.

MRE has recently emerged as a noninvasive imaging technique for assessing the microstructural integrity of the brain (e.g., [35,36]). MRE is sensitive to both the microstructural composition (i.e., stiffness) and the microstructural organization (i.e., damping ratio) of brain tissue [37,38,39]. Previous work using MRE has demonstrated that brain stiffness decreases across many neurodegenerative conditions including multiple sclerosis (e.g., [40]), amyotrophic lateral sclerosis (e.g., [41]), mild cognitive impairment (e.g., [42]), and Alzheimer’s disease (e.g., [43]).

MRE-derived stiffness measures have also been shown to decline across the lifespan (for reviews see [44,45,46]). Indeed, Sack and colleagues [47,48] reported that whole-brain stiffness declines at a rate three times greater than brain volume with an estimated 0.8% change annually. Recent advances in MRE image resolution have permitted researchers to assess age-related changes in individual anatomical structures. For example, Hiscox and colleagues [49] have reported age differences in stiffness in subcortical structures (e.g., caudate, putamen) when comparing groups of young and older adults. These data again highlight the sensitivity of MRE as a marker of early stages of age-related change. In addition to being sensitive to age-related changes in tissue stiffness, MRE-derived measures of microstructural integrity are also sensitive to cognitive performance outcomes in related anatomical regions among both young (e.g., [50,51,52]) and older (e.g., [53,54,55]) adults. We propose that MRE may reveal that basal ganglia integrity is indeed related to implicit sequence learning outcomes when volumetric measures fail to show such a relationship.

In the current study, healthy middle-aged and older adults completed both an MRI and MRE scan as well as the SRTT to assess sequence learning and a Generation Task to assess implicit vs. explicit sequence knowledge. The goal was to evaluate the relationships between age-related implicit learning performance and both volume and mechanical properties of the striatum highlighting the specificity of the structure–function relationship. The putamen was the primary focus due to its role in implicit sequence learning specifically [56,57,58] and the caudate was also evaluated for its role in sequence learning more generally [4,59].

## 2. Method

### 2.1. Participants

A total of 61 cognitively healthy middle-aged and older adult volunteers (45–78 years old, mean = 61.5 years; *SD* = 10.4, 23 males, 38 females) participated in this study. Participants were recruited from the Champaign-Urbana community. All participants provided informed consent, and study procedures were approved by the University of Illinois at Urbana-Champaign Institutional Review Board. Primary outcome measures were performance on the SRTT (described below), putamen and caudate volumes (measured via structural MRI; Siemens Medical Solutions, Erlangen, Germany), and putamen and caudate stiffness and damping ratio (measured via Magnetic Resonance Elastography, Resoundant, Rochester, MN, USA, also described below). Data from four participants were excluded due to equipment failure (N = 1), behavioral measures (described below) greater than 3 standard deviations above the mean (N = 2), and putamen stiffness measures greater than 3 standard deviations below the mean (N = 1). The final data set included 57 participants (22 males, 35 females; ages 45–78 years old, mean = 61.7, SD = 10.2, 78.9% white). Also, the sample was highly educated, all participants had some post-secondary education with a mean of 17.5 years (SD = 2.2) of education, indicative of educational attainment beyond a college degree.

All participants also completed a standard neuropsychological battery of tasks to capture a snapshot of cognitive health in the sample. This battery included the Montreal Cognitive Assessment (MoCA; [60]), delayed word recall from the California Verbal Learning Test (CVLT; [61]), and the digit-symbol substitution task [62]. Mean MoCA score was 27.7 (SD = 1.95), mean word recalled after a delay on the CVLT was 12.5 out of 16 (SD = 2.9), and mean number of accurate digit-symbol substitutions was 57.2 (SD = 10.4).

### 2.2. Stimuli and Apparatus

Participants sat approximately 60 cm from a 21” LCD computer monitor (Dell Inc., Round Rock, TX, USA) in a private, quiet room. Stimuli were presented using either E-prime 2.0 (for the SRTT) or E-prime 3.0 (for the Generate Task) software [63] on a desktop computer (Dell Inc., Round Rock, TX, USA, 1024 × 768 resolution). Responses were made using the Z-key and X-key with the left hand and period-key and forward-slash-key with the right hand. The stickers were placed on the response keys so that participants knew easily where to place their hands.

Stimuli were four evenly spaced annuli presented horizontally in the center of the screen. The annuli were white on a black background. Each annulus subtended 3.2° × 3.2° of visual angle. The entire stimulus display subtended 22.5° of visual angle horizontally and 3.2° of visual angle vertically.

### 2.3. Stimulus Sequences

Two second-order conditional sequences consistent with the statistical rules outlined by Reed and Johnson [64] were used in this study. For each participant, one sequence (e.g., Sequence A) was used in blocks 1–8 and 10. This was the learned sequence. The other sequence (e.g., Sequence B) was used on block 9 (i.e., the Transfer Block) to test learning of the studied sequence. Which sequence was used on study blocks vs. the transfer block was counterbalanced across participants.

### 2.4. Design and Procedure

#### 2.4.1. Serial Reaction Time Task

On each trial, participants were presented with four white, evenly spaced annuli positioned horizontally across the screen for 500 ms. Then one of the annuli was filled in (white). The shaded circle served as the target stimulus and participants indicated its location by pressing one of four buttons each mapped to the four annulus positions (i.e., left middle finger for the left-most target, left index finger for the left inner target, right index finger for the right inner target, and right middle finger for the right-most target). The next trial began as soon as a response was made. There were 96 trials per block and a total of 10 blocks. At the end of every block, block accuracy and average reaction time were presented in white font at the center of the black screen. Participants were also informed how many blocks were left to complete at this time.

Participants watched video instructions prior to the start of the task. The video showed participants what the stimuli looked like, how they should position their hands, and which button to use to respond to each of the four target locations. Participants were told the goal of the task was to respond as quickly and accurately as possible when each stimulus appeared on the computer screen. Participants were not told about the underlying sequence. Participants practiced the task prior to completing the experimental blocks. Practice included a single block with 20 trials. Trials were identical to the experimental trials except that the stimulus order did not follow a repeating sequence.

#### 2.4.2. Generate Task

The Generation Task is a standard test used to assess levels of implicit vs. explicit sequence knowledge [65]. After the SRTT was completed, participants were told that the targets had followed a repeating pattern or sequence throughout the task and that they would complete an additional task to assess whether they had become aware of the sequence. They then completed two separate Generation Task blocks. On each generation block, participants again saw four, evenly spaced white annuli positioned horizontally across the screen. The response mapping was identical to the SRTT mapping. Participants would then hit a button, and the corresponding annulus was filled in (white). Participants were instructed to make 26 button pushes and were not permitted to press the same button twice in a row. During the first Generation Task block, participants were told to try to recreate the sequence that they had learned during the SRTT (i.e., Include Instructions). During the second Generation Task block, participants were told to NOT recreate the sequence they had learned and instead create a brand-new sequence (i.e., Exclude Instructions).

### 2.5. Behavioral Outcome Measures

#### 2.5.1. Serial Reaction Time Task

Reaction time (RT) data were inspected for all subjects. Occasionally, participants paused to ask a question or take a drink at an inappropriate time resulting in very long RTs, these RTs (all greater than 20 s) were excluded from the analysis. There were 11 such trials across all blocks and all participants. Block accuracy and average block reaction time for correct trials were calculated separately for each block. To assess sequence acquisition, a Learning Score was computed for each participant. Learning Score was calculated as the RT improvement across the first 8 sequenced blocks. To assess sequence learning, a Transfer Score was calculated for each participant. The Transfer Score was the difference in RT between Transfer Block 9 and the surrounding blocks (i.e., Blocks 8 and 10). This is the standard measure of implicit sequence learning in the SRTT literature (for review see [13]). The data file for one block (block 1) for one participant was corrupted, could not be restored. That participant was excluded from the Learning Score analysis.

#### 2.5.2. Generation Task

The Process Dissociation Procedure Generation Task was analyzed as in Destrebeqz and Cleeremans to determine explicit vs. implicit sequence knowledge [65]. The number of generated three-element chunks that were part of the studied training sequence was calculated independently for the include and exclude instructions. Because the generated sequence was 26 positions long, the maximum number of correct chunks was 24. The generation task score was computed by dividing the number of chunks generated consistent with the study sequence by 24. Because participants were not allowed to press the same button sequentially, the chance was 33%. Seven participants were excluded from the Generation Task analysis due to file corruption (N = 3), failure to follow instructions (N = 1; i.e., only hit one button), and failure to follow exclude task instructions (N = 3).

### 2.6. MRI Apparatus

Imaging data were acquired using a Siemens 3T Prisma whole-body MRI scanner (Siemens Medical Solutions, Erlangen, Germany) and standard 64-channel radio-frequency head coil (Siemens Medical Solutions, Erlangen, Germany). Foam padding was used to limit head motion. Data were collected at the Biomedical Imaging Center at the Beckman Institute for Advanced Science and Technology at the University of Illinois at Urbana-Champaign.

### 2.7. Volume Acquisition and Processing

A T_1_-weighted MPRAGE sequence was used to acquire structural images from each participant. Images were acquired with 0.8 mm^3^ isotropic resolution (TR = 2500 ms; TE = 2.22 ms; TI = 1000 ms; Flip angle = 8°). The recon-all pipeline and Desikane-Killiany Atlas from Freesurfer v. 7.2 [66] were used to extract bilateral putamen, caudate, and amygdala volumes. Intracranial volume was also extracted and used to normalize [67] each ROI for head size (e.g., [27,33]). Regional volumes were converted to anatomical binary masks for the MRE analysis.

### 2.8. MRE Acquisition and Processing

**Figure 1:**
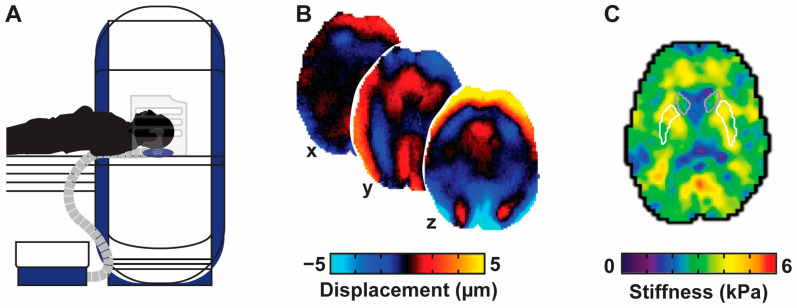
Overview of the high-resolution MRE protocol used in the current study. (**A**) Actuation: Vibrations are delivered via a soft head pillow pneumatic driver while participants lay in the MRI scanner generating shear waves in the tissue. (**B**) Imaging: Shear waves are imaged in x, y, and z dimensions using a high-resolution MRE sequence. (**C**) Segmentation and inversion: Freesurfer segmentations are converted to anatomical masks and nonlinear inversion algorithms are used to calculate shear stiffness in those regions. White and gray outlines provide illustrative indications of putamen and caudate locations, respectively.

Our standard elastography procedure has been reported in detail previously [51]. A small, soft pneumatic actuator (Resoundant, Rochester, MN, USA) was placed behind the participant’s head as they lay supine in the MRI scanner (Siemens Medical Solutions, Erlangen, Germany; Figure 1A). During MRE acquisition, the actuator vibrated the head at 50 Hz. A 3-D multiband, multishot spiral MRE sequence [68,69] for capturing high-resolution displacement data was used to encode the resulting tissue deformation from the vibration [70]. Motion was captured along three separate axes via repeating motion-sensitive gradients embedded in the sequence (Figure 1B). Displacement fields were sampled at four points across one period of vibration. Imaging parameters were 240 mm × 240 mm field of view, 150 × 150 imaging matrix, 72 slices, 1.6 mm slice thickness, TR/TE = 2520/75 ms. MRE data were processed in native space. A nonlinear inversion algorithm [71] was used to calculate tissue property measures using anatomical masks of regions-of-interest as spatial priors through soft prior regularization [72]. Masks were created in native space by registering the T_1_-weighted images to MRE magnitudes using FLIRT in FSL v. 5.0.7 [73,74], and applying the transform to binary anatomical masks from Freesurfer. Nonlinear inversion estimates the complex shear modulus, $G\;=\;G^{\prime }+iG^{\prime \prime }$ ($G^{\prime }$ is the storage modulus and $G^{\prime \prime }$ is the loss modulus), which was used to compute the viscoelastic shear stiffness, $\mu \;=\;\frac{{2|G|}^{2}}{G^{\prime }+|G|}$ (Figure 1C) and damping ratio, $\xi \;=\;\frac{G^{\prime \prime }}{{2G}^{\prime }}$. We have previously shown that this approach is highly reliable and that in an assessment of test–retest reliability, the coefficient of variation for putamen stiffness and damping ratio was 2.4% and 4.2%, respectively, and the coefficient of variation for caudate stiffness and damping ratio was 3.4% and 3.7%, respectively [75].

### 2.9. Regions of Interest and Neuroimaging Outcome Measures

Both striatal structures were considered. The putamen was the primary region of interest because of its known relationship with implicit sequence learning specifically [56,57]. The caudate was the secondary region of interest due to its known relationship with both implicit and explicit sequence learning [4,59]. Volume, stiffness, and damping ratio were assessed for both striatal structures. Bilateral amygdala stiffness was also included as a control region.

### 2.10. Statistical Analyses

Sequence learning was assessed in two ways. First, a repeated measures ANOVA across the first 8 sequenced blocks was conducted separately for accuracy and RT. Next, sequence learning was assessed via paired-samples t-test comparing RT on transfer block 9 to the average RT on the surrounding sequence blocks 8 and 10. A Transfer Score (i.e., the difference in RT between block 9 and the average of blocks 8 and 10) was then computed and used in subsequent analyses. To evaluate explicit sequence knowledge, the number of generated three-position chunks, in the generate task, that were consistent with the learned sequence, was calculated and divided by the total number of three-position chunks generated (i.e., [65]). This was carried out separately for both inclusion and exclusion instructions and those scores were evaluated against chance using one-tailed, one-sample *t*-tests as is standard for the Process Dissociation Procedure [56,65]. The relationship between age and outcome measures of interest (i.e., Transfer Score and structural measures in the caudate and putamen) was evaluated with bivariate correlations. The relationship between Transfer Score and both putamen and caudate structural measures (i.e., volume, stiffness, and damping ratio) was evaluated with partial correlations controlling for age and sex. Distribution of residuals for each of the variables of interest was assessed for normality with the Kolmogorov–Smirnov test. None of the distributions were found to be non-normally distributed (*p* > 0.05 in all cases). Finally, control analyses were performed to assess the specificity of the putamen stiffness–sequence learning relationship. Partial correlations controlling for age and sex were performed comparing amygdala stiffness and Transfer Scores as well as for putamen stiffness and Delayed Recall Memory scores. Significance was evaluated at *p* < 0.05. Corrections for sphericity violations were performed where appropriate.

## 3. Results

### 3.1. Implicit Sequence Learning

**Figure 2:**
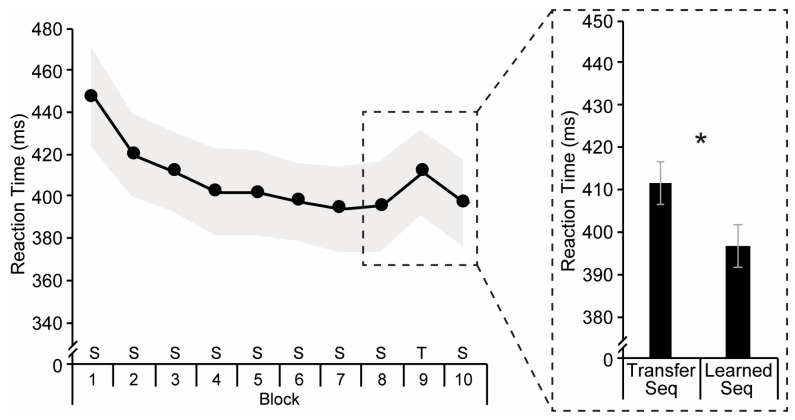
Average reaction times across the ten experimental blocks. Gray shading indicates a 95% confidence interval. S = learned sequence; T = transfer sequence. The inset on the right shows reaction times for the transfer sequence (i.e., Block 9) and the average reaction time of the surrounding learned sequence blocks (i.e., Blocks 8 and 10). Error bars show the standard error of the difference. Asterisk indicates a significant difference between conditions.

Changes in accuracy across the eight sequence learning blocks were assessed with a repeated measures ANOVA. The main effect of block was significant, *F*(5.8, 325.4) = 1.54, *p* = 0.168, *η_p_*^2^ = 0.03, indicating that accuracy was stable across learning (average accuracy = 98.0%). Implicit sequence learning was assessed in two ways: Learning Score and Transfer Score. Learning Score was measured by identifying RT improvement across the eight learning blocks which was assessed with a repeated measures ANOVA (Figure 2). The main effect of Block, *F*(5.3, 299.0) = 16.20, *p* < 0.001, *η_p_*^2^ = 0.22, was significant indicating that participants became faster across the first eight sequenced blocks, suggesting that they had acquired sequence knowledge. Transfer Score was assessed with a paired-samples *t*-test (Figure 2). The Transfer Score was significant, *t*(56) = 3.04, *p* = 0.004, *d* = 0.40, a second indicator that participants did, indeed, learn the implicit sequence. As Transfer Scores are a more pure measure of sequence learning independent of practice effects [13], Transfer Score was included in all subsequent analyses as our measure of implicit sequence learning.

### 3.2. Explicit Sequence Knowledge

**Figure 3:**
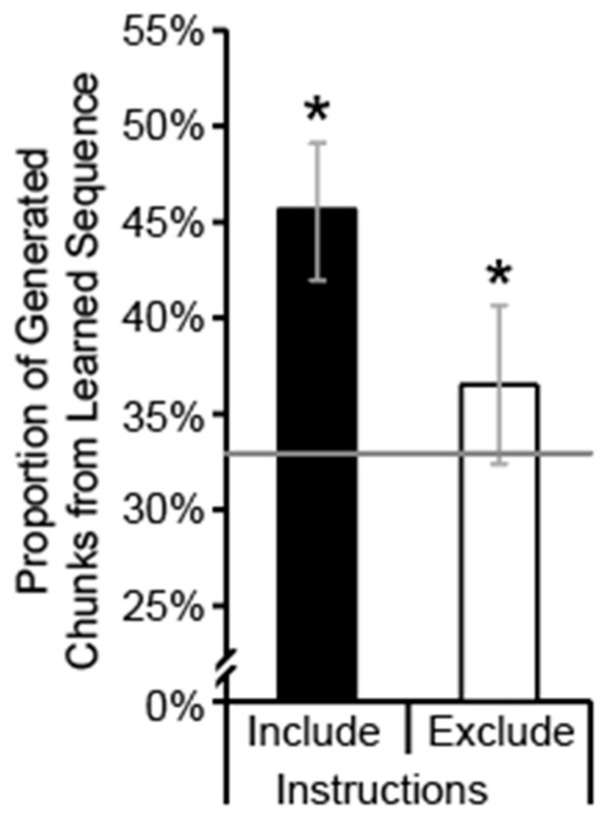
Proportion of generated 3-position chunks that were part of the learned sequence under inclusion instructions (black bars) and exclusion instructions (white bars) in the Generation Task. Error bars are 95% confidence intervals. The gray line indicates chance. Asterisks indicate a significant difference compared to chance.

Explicit sequence knowledge was assessed using the Process Dissociation Procedure Generation Task [65]. Under inclusion instructions, 45.5% (SD = 13.1%) of the chunks generated were consistent with the studied sequence and this was above chance (33%), *t*(49) = 6.75, *p* < 0.001, *d* = 0.96. Under exclusion instructions, 36.5% (SD = 14.6%) of the chunks generated were consistent with the studied sequence and this was not significantly different from chance, *t*(49) = 1.71, *p* = 0.047, *d* = 0.24 (Figure 3). These data suggest that, as a group, participants did not have explicit sequence knowledge and were unable to control when they did and did not generate chunks from the learned sequence [65].

### 3.3. Effects of Age

**Figure 4:**
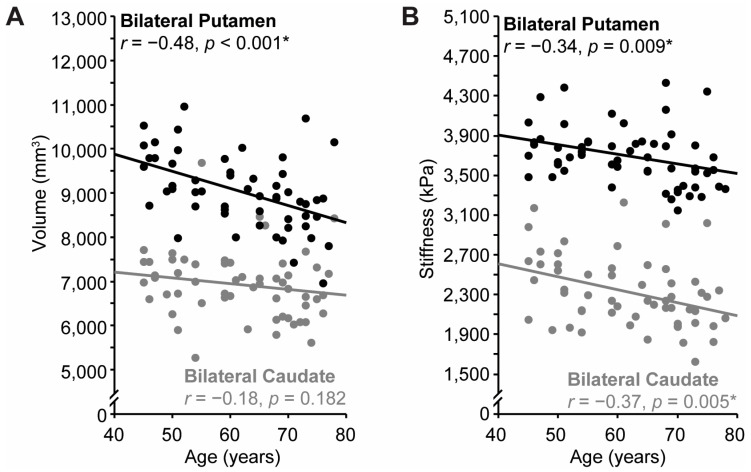
Relationship between age and (**A**) bilateral putamen (black) and caudate (gray) volume and (**B**) bilateral putamen (black) and caudate (gray) stiffness. Regression lines shown. Asterisks indicate a statistically significant correlation.

The relationship between age and our various measures of interest (i.e., Transfer Score and putamen/caudate volume, stiffness, and damping ratio) was assessed via bivariate correlation. Age was significantly negatively correlated with both bilateral putamen volume, *r* = −0.48, *p* < 0.001 (Figure 4A, black circles), and bilateral putamen stiffness, *r* = −0.34, *p* = 0.009 (Figure 4B, black circles), indicating age-related decline, but not bilateral putamen damping ratio, *r* = 0.04, *p* = 0.798. Age was significantly correlated with bilateral caudate stiffness, *r* = −0.37, *p* = 0.005 (Figure 4B, gray circles), but not bilateral caudate volume, *r* = −0.18, *p* = 0.182 (Figure 4B, gray circles), or bilateral caudate damping ratio, *r* = 0.04, *p* = 0.766. As in previous MRE investigations [47,76,77], annual stiffness change was computed. Putamen stiffness decreased 0.010 kPa per year and caudate stiffness decreased 0.013 kPa per year. Age was not correlated with Transfer Score (*r* = 0.10, *p* = 0.469) in this sample.

### 3.4. Relationship Between Striatal Structure and Sequence Learning

**Figure 5:**
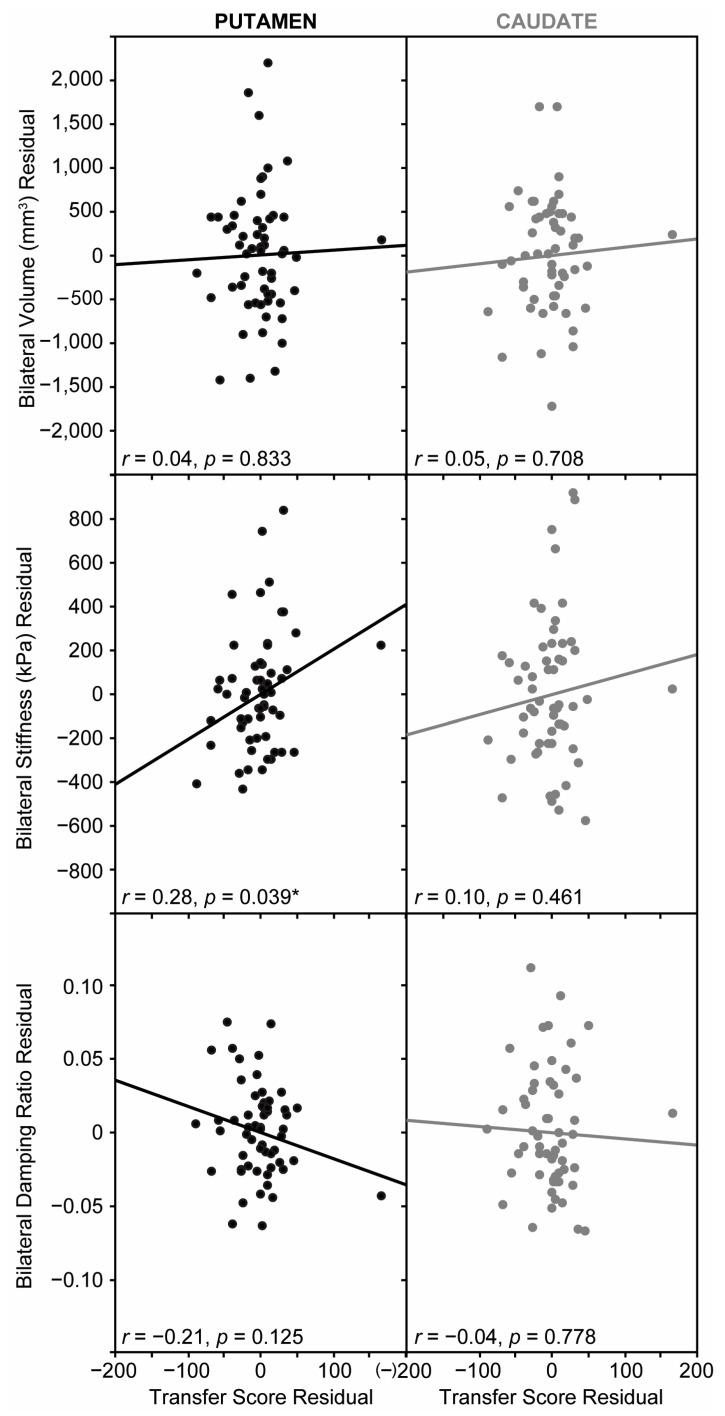
(**left**) Transfer score residuals are plotted against bilateral putamen volume residuals (**top**), bilateral putamen stiffness residuals (**middle**), and bilateral putamen damping ratio residuals controlling for age and sex. (**right**) Transfer score residuals are plotted against bilateral caudate volume residuals (**top**), bilateral caudate stiffness residuals (**middle**), and bilateral caudate damping ratio residuals controlling for age and sex. Regression line shown. Asterisk indicates a statistically significant correlation.

To examine the relationship between striatal structure and sequence learning outcomes, partial correlations controlling for age and sex and comparing Transfer Score with putamen and caudate integrity were conducted independently for volume, stiffness, and damping ratio (Figure 5). Putamen structure was our primary region of interest. The relationship between putamen volume and Transfer Score was not significant, *r* = 0.04, *p* = 0.833 nor was the relationship between putamen damping ratio and Transfer score, *r* = −0.21, *p* = 0.125. The relationship between putamen stiffness and Transfer Score was significant, *r* = 0.28, *p* = 0.039, indicating that individuals with greater putamen stiffness had higher scores of successful sequence learning. None of the relationships between Transfer Score and caudate volume (*r* = 0.05, *p* = 0.708), nor caudate stiffness (*r* = 0.10, *p* = 0.461), nor caudate damping ratio (*r* = −0.04, *p* = 0.778), was significant.

### 3.5. Control Analyses

**Figure 6:**
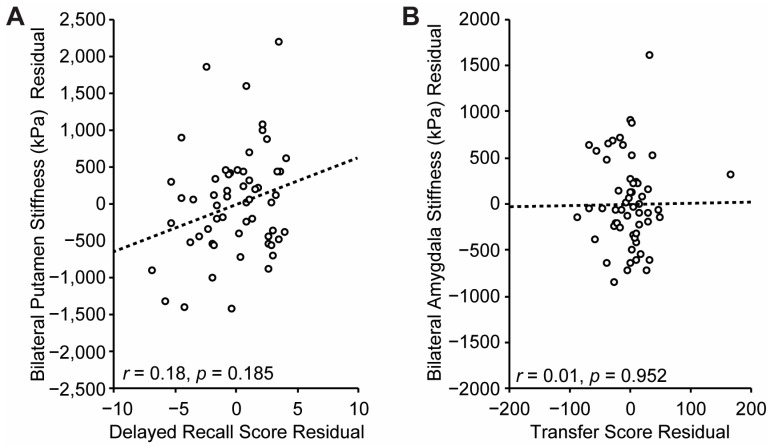
Data from the control analyses plotted. (**A**) Delayed Word List Recall Score residuals from the California Verbal Learning Test plotted against bilateral putamen stiffness controlling for age and sex. (**B**) Transfer score residuals are plotted against bilateral amygdala stiffness after controlling for age and sex. Regression line shown.

To assess the specificity of this relationship, two control analyses were also performed. First, to assess the specificity of the putamen–sequence learning relationship, putamen stiffness was correlated (again controlling for age and sex) with performance on the delayed word recall task from the CVLT. Delayed word recall was not expected to depend heavily on putamen processes, as it is a standard neuropsychological test of hippocampal function (e.g., [31]). As anticipated, putamen stiffness and Delayed Recall Memory scores were not significantly correlated, *r* = 0.18, *p* = 0.185 (Figure 6A). Second, to further evaluate the specificity of the putamen–sequence learning relationship, Transfer Scores were correlated (again controlling for age and sex) with amygdala stiffness, a subcortical region involved in emotional processing and not thought to be differentially involved in implicit sequence learning. One participant was excluded because their amygdala stiffness was 3 standard deviations above the mean. The relationship between amygdala stiffness and Transfer Score was also not significant, *r* = 0.01, *p* = 0.952 (Figure 6B).

## 4. Discussion

Few studies have investigated the relationship between implicit motor sequence learning outcomes and the structure of the brain. However, there are many reasons to hypothesize that the structure of sequence learning relevant regions should be related to performance outcomes on a sequence learning test. We proposed that one reason for a lack of such reports is that traditional structural MRI measures, like volumetry, may not be sufficiently sensitive to capture more subtle structural features that may be meaningfully related to sequence learning success and failures. In this study, we took advantage of the enhanced sensitivity of MRE for identifying microstructural variability in brain stiffness, to explore these relationships.

Our work focused on the structures of the striatum as the primary hub of sequence learning in the brain (for review see [4]) with a particular focus on the putamen, which functional imaging data suggests supports implicit learning specifically [56,57,58]. We found that the putamen becomes less stiff with increased age. Further, as predicted, putamen stiffness, but not volume, correlated with sequence learning outcomes on the SRTT. Stiffness in the caudate, a region more generally associated with both implicit and explicit sequence knowledge, did not correlate with Transfer score highlighting the specificity of the putamen relationship. Together these data suggest that MRE-derived measures of stiffness may be a more sensitive measure of putamen integrity than traditional volumetric measures and more sensitive to the relationship between putamen integrity and sequence learning success. The specificity of this relationship was further probed with a series of control analyses. As anticipated, putamen stiffness did not correlate with a task unrelated to the putamen (i.e., delayed word memory) and sequence learning scores did not correlate with stiffness in a region unrelated to sequence learning (i.e., amygdala). These data support the notion that when the appropriately sensitive tools are applied, expected structure-function relationships are uncovered.

This study adds to a growing body of literature suggesting that MRE is a powerful tool for identifying specific structure–function relationships. While all of the existing studies comparing MRE measures of structural integrity and cognitive outcomes have focused on the hippocampus and its subfields [51,53] or regions of the cortex [50,55], this is the first study to investigate such relationships in the striatum, providing an important additional step in exploring the global utility of MRE for cognitive neuroscience investigations. Importantly, perhaps, the current findings provide a conceptual replication of other published studies investigating such structure–function relationships using MRE in aging adults. Hiscox and colleagues [54] were the first to examine structure–function relationships in healthy older adults using MRE, combining region-specific measures of mechanical properties with sensitive cognitive tasks. In a very small sample (N = 11; ages 65–75), they showed that older adults with better left hippocampal integrity performed better on an immediate recall of a verbal paired-associates task. Delgorio and colleagues [53] extended this work in a larger sample (N = 88; ages 23–81; mean age = 60), demonstrating that hippocampal subfield stiffness (CA1-2, DG-CA3, and subiculum assessed separately) all positively correlated with performance on both a delayed story recall task and a spatial memory task. These data indicate that, as in the current paper, aging individuals with higher regional stiffness also perform better on cognitive tasks associated with that specific anatomical region.

Perhaps of note, in all MRE investigations exploring the relationship between cognitive processes and the mechanical properties of associated anatomical regions among young adults (e.g., [50,51]), significant correlations are reported in damping ratio, but not stiffness measures. The current findings in a group of older adults found the opposite (i.e., a significant relationship with stiffness, but not damping ratio). Damping ratio and stiffness are typically considered to be associated with different aspects of tissue microstructure (for review see [45]). Damping ratio measures reflect the attenuation of vibration through tissue, which is affected by the geometric organization of the cells within the tissue [38,78]. Stiffness measures, rather, reflect the cellular composition of tissue and are sensitive to both neurodegeneration [79] and demyelination [39]. The current data are also consistent with other recent work [53] that found that in a lifespan sample (ages 23–81) that stiffness of hippocampal subfields was associated with performance on different memory tasks. Future work is necessary to probe this possible dissociation with changes in stiffness being more associated with cognitive outcomes in older populations and differences in damping ratio being more associated with cognitive outcomes in young adults.

The current work also extends the general MRE and aging findings to an additional anatomical region. Interestingly, in the growing number of MRE studies investigating the relationship between aging and brain stiffness, the results thus far have been remarkably consistent despite differences in the acquisition approach and regions of interest. As noted earlier, in an early investigation of the stiffness–age relationship, Sack and colleagues [48] reported a 0.015 kPa reduction in stiffness per year in a large, central, cerebral slab of the brain. Arani and colleagues [76] performed a similar investigation of the effect of age across all lobes of the cerebrum identifying a negative correlation in all lobes and estimated a stiffness decrease of 0.011 kPa per year. Delgorio and colleagues [77] were the first to look at the relationship between age and stiffness in individual anatomical structures focusing on the hippocampal subfields. They found an annual decrease in stiffness of 0.011 kPa in each of the DG-CA3 region, CA1-CA2 region, and subiculum as well as a 0.014 kPa decrease in the entorhinal cortex. In the current study, we show an annual decrease of 0.010 kPa and 0.013 kPa per year for putamen and caudate stiffness, respectively. Thus, there is considerable consistency in those reported investigations thus far.

This study was not without its limitations. Our sample was very highly educated, which may limit the generalizability of these findings. All participants had some post-secondary education with a mean of 17.5 years of education, indicating that, on average, our participants had some educational attainment beyond college. Higher educational attainment has been associated with greater cognitive reserve in older adult populations, which can facilitate cognitive performance through multiple or optimal strategic approaches when completing cognitive tasks [80]. Higher education has also been associated with preserved putamen function among patients with Parkinson’s disease [81]. Together these findings suggest that education may be contributing to the lack of correlation between Transfer Score and age and also may be minimizing the relationship between putamen structural measures and Transfer Effects. Future studies should prioritize a more educationally diverse sample more representative of the general population.

Our sample was also 78.9% white. While considerable efforts were made to contact a more diverse participant pool, these data were collected in 2020 and 2021 during the SARS-COVID-2 pandemic and many potential participants were understandably reticent to participate in an in-person research study despite the extensive efforts that were made to adhere to local and federal guidelines at the time. Our sample was also disproportionately female (61.4%). Previous studies have identified sex differences in MRE stiffness in various brain regions [47,76]. As our sample was not large enough to report sex-specific effects reliably, we controlled for sex in the relevant analyses.

Finally, this study was very tightly focused on two specific regions, the caudate which is known to be important for sequence learning generally, and the putamen, which is known to be important for implicit sequence learning specifically. This was a principled approach based on the existing functional MRI studies, structural lesion studies, and patient studies. Previous studies, however, have demonstrated that as implicit sequence knowledge becomes explicit, working memory processes as well as goal-specific control processes come online, resulting in dorsolateral prefrontal activation [58,59,82]. Future investigations should consider extending the current findings by evaluating the relationship between anatomical structural integrity in implicit and explicit sequence learning. The current study design was not optimized for this approach as there was no explicit condition and participants did not demonstrate explicit sequence knowledge. Additionally, imaging parameters were not optimized to assess dorsolateral cortical structures using MRE. As MRE is an emerging tool in the study of brain health and brain dynamics, research continues assessing how best to optimize sequence acquisition parameters and analysis parameters for various unique anatomical structures. The current study used the optimized subcortical gray matter protocol outlined by our group [75] that has been used successfully in identifying relationships between memory and hippocampal microstructure (e.g., [51,83]). Accurate imaging of cortical structures has several unique challenges. For example, individual cortical structures are thinner and more geometrically complex than subcortical structures, both of which are exacerbated in age. Hiscox and colleagues [84] have recently outlined a series of guidelines for successful and accurate cortical MRE, highlighting the importance of increased spatial resolution of the image acquisition (i.e., 1.25 mm isotropic resolution) as well as changes to various analysis parameters. Indeed, data is emerging showing that this approach is fruitful for identifying relationships between regional structural integrity and personality traits [85]. Future work should prioritize this approach for investigating the relationship between brain structure and cognitive processes as well.

## 5. Conclusions

In conclusion, this is the first report using MRE to investigate the relationship between the microstructure of the striatum and cognitive performance on a task that depends critically on those structures. This report adds to a small but growing literature highlighting the potential for MRE as a useful tool in studying structure–function relationships when traditional tools, like volumetry, have failed to report such theoretically strong relationships. Further, this report highlights the utility of MRE in studying the microstructural health of the aging brain in healthy populations, with promise for significant utility in clinically affected populations as well.

## Data Availability

The data presented in this study are available on request from the corresponding author due to University of Illinois at Urbana-Champaign IRB restrictions related to studies where participants did not explicitly consent to have their data shared in a public repository.

## Abbreviations

The following abbreviations are used in this manuscript:

MRE	Magnetic Resonance Elastography
SRTT	Serial Reaction Time Task
RT	Reaction Time
CVLT	California Verbal Learning Test

## References

[1] Koch F.-S., Sundqvist A., Thornberg U.B., Nyberg S., Lum J.A.G., Ullman M.T., Barr R., Rudner M., Heimann M. (2020). Procedural Memory in Infancy: Evidence from Implicit Sequence Learning in an Eye-Tracking Paradigm. J. Exp. Child Psychol..

[2] Frensch P.A., Miner C.S. (1994). Effects of Presentation Rate and Individual Differences in Short-Term Memory Capacity on an Indirect Measure of Serial Learning. Mem. Cognit..

[3] Howard J.H., Howard D.V. (1997). Age Differences in Implicit Learning of Higher Order Dependencies in Serial Patterns. Psychol. Aging.

[4] Janacsek K., Shattuck K.F., Tagarelli K.M., Lum J.A.G., Turkeltaub P.E., Ullman M.T. (2020). Sequence Learning in the Human Brain: A Functional Neuroanatomical Meta-Analysis of Serial Reaction Time Studies. NeuroImage.

[5] Freidle M., Thompson W.H., Albrecht F., Franzén E. (2023). Implicit Motor Sequence Learning in People with Mild to Moderate Parkinson’s Disease: Behavior and Related Brain Function. J. Park. Dis..

[6] Clark G.M., Lum J.A.G., Ullman M.T. (2014). A Meta-Analysis and Meta-Regression of Serial Reaction Time Task Performance in Parkinson’s Disease. Neuropsychology.

[7] Jackson G.M., Jackson S.R., Harrison J., Henderson L., Kennard C. (1995). Serial Reaction Time Learning and Parkinson’s Disease: Evidence for a Procedural Learning Deficit. Neuropsychologia.

[8] Kim J.-S., Reading S.A.J., Brashers-Krug T., Calhoun V.D., Ross C.A., Pearlson G.D. (2004). Functional MRI Study of a Serial Reaction Time Task in Huntington’s Disease. Psychiatry Res. Neuroimaging.

[9] Knopman D., Nissen M.J. (1991). Procedural Learning Is Impaired in Huntington’s Disease: Evidence from the Serial Reaction Time Task. Neuropsychologia.

[10] Raz N., Rodrigue K.M., Kennedy K.M., Head D., Gunning-Dixon F., Acker J.D. (2003). Differential Aging of the Human Striatum: Longitudinal Evidence. AJNR Am. J. Neuroradiol..

[11] Nissen M.J., Bullemer P. (1987). Attentional Requirements of Learning: Evidence from Performance Measures. Cognit. Psychol..

[12] Clegg B.A., DiGirolamo G.J., Keele S.W. (1998). Sequence Learning. Trends Cogn. Sci..

[13] Schwarb H., Schumacher E.H. (2012). Generalized Lessons about Sequence Learning from the Study of the Serial Reaction Time Task. Adv. Cogn. Psychol..

[14] Cohen A., Ivry R.I., Keele S.W. (1990). Attention and Structure in Sequence Learning. J. Exp. Psychol. Learn. Mem. Cogn..

[15] Grafton S.T., Hazeltine E., Ivry R.B. (1998). Abstract and Effector-Specific Representations of Motor Sequences Identified with PET. J. Neurosci. Off. J. Soc. Neurosci..

[16] Deroost N., Coomans D. (2018). Is Sequence Awareness Mandatory for Perceptual Sequence Learning: An Assessment Using a Pure Perceptual Sequence Learning Design. Acta Psychol..

[17] Willingham D.B., Nissen M.J., Bullemer P. (1989). On the Development of Procedural Knowledge. J. Exp. Psychol. Learn. Mem. Cogn..

[18] Cherry K.E., Stadler M.A. (1995). Implicit Learning of a Nonverbal Sequence in Younger and Older Adults. Psychol. Aging.

[19] Bennett I.J., Howard J.H., Howard D.V. (2007). Age-Related Differences in Implicit Learning of Subtle Third-Order Sequential Structure. J. Gerontol. B. Psychol. Sci. Soc. Sci..

[20] Howard D.V., Howard J.H., Japikse K., DiYanni C., Thompson A., Somberg R. (2004). Implicit Sequence Learning: Effects of Level of Structure, Adult Age, and Extended Practice. Psychol. Aging.

[21] Doyon J., Gabitov E., Vahdat S., Lungu O., Boutin A. (2018). Current Issues Related to Motor Sequence Learning in Humans. Curr. Opin. Behav. Sci..

[22] Penhune V.B., Steele C.J. (2012). Parallel Contributions of Cerebellar, Striatal and M1 Mechanisms to Motor Sequence Learning. Behav. Brain Res..

[23] Sefcsik T., Nemeth D., Janacsek K., Hoffmann I., Scialabba J., Klivenyi P., Ambrus G.G., Haden G., Vecsei L. (2009). The Role of the Putamen in Cognitive Functions—A Case Study. Learn. Percept..

[24] Boyd L.A., Edwards J.D., Siengsukon C.S., Vidoni E.D., Wessel B.D., Linsdell M.A. (2009). Motor Sequence Chunking Is Impaired by Basal Ganglia Stroke. Neurobiol. Learn. Mem..

[25] Vakil E., Kahan S., Huberman M., Osimani A. (2000). Motor and Non-Motor Sequence Learning in Patients with Basal Ganglia Lesions: The Case of Serial Reaction Time (SRT). Neuropsychologia.

[26] Raz N., Gunning-Dixon F., Head D., Rodrigue K.M., Williamson A., Acker J.D. (2004). Aging, Sexual Dimorphism, and Hemispheric Asymmetry of the Cerebral Cortex: Replicability of Regional Differences in Volume. Neurobiol. Aging.

[27] Raz N., Lindenberger U., Rodrigue K.M., Kennedy K.M., Head D., Williamson A., Dahle C., Gerstorf D., Acker J.D. (2005). Regional Brain Changes in Aging Healthy Adults: General Trends, Individual Differences and Modifiers. Cereb. Cortex.

[28] Elkattan A., Mahdy A., Eltomey M., Ismail R. (2017). A Study of Volumetric Variations of Basal Nuclei in the Normal Human Brain by Magnetic Resonance Imaging. Clin. Anat..

[29] Halkur Shankar S., Ballal S., Shubha R. (2017). Study of Normal Volumetric Variation in the Putamen with Age and Sex Using Magnetic Resonance Imaging. Clin. Anat..

[30] Rodrigue K.M., Kennedy K.M. (2011). The Cognitive Consequences of Structural Changes to the Aging Brain. Handbook of the Psychology of Aging.

[31] Petersen R.C., Jack C.R., Xu Y.-C., Waring S.C., O’Brien P.C., Smith G.E., Ivnik R.J., Tangalos E.G., Boeve B.F., Kokmen E. (2000). Memory and MRI-Based Hippocampal Volumes in Aging and AD. Neurology.

[32] Gunning-Dixon F.M., Raz N. (2003). Neuroanatomical Correlates of Selected Executive Functions in Middle-Aged and Older Adults: A Prospective MRI Study. Neuropsychologia.

[33] Head D., Kennedy K.M., Rodrigue K.M., Raz N. (2009). Age Differences in Perseveration: Cognitive and Neuroanatomical Mediators of Performance on the Wisconsin Card Sorting Test. Neuropsychologia.

[34] Raz N., Williamson A., Gunning-Dixon F., Head D., Acker J.D. (2000). Neuroanatomical and Cognitive Correlates of Adult Age Differences in Acquisition of a Perceptual-Motor Skill. Microsc. Res. Tech..

[35] Murphy M.C., Huston J., Ehman R.L. (2019). MR Elastography of the Brain and Its Application in Neurological Diseases. NeuroImage.

[36] Muthupillai R., Lomas D.J., Rossman P.J., Greenleaf J.F., Manduca A., Ehman R.L. (1995). Magnetic Resonance Elastography by Direct Visualization of Propagating Acoustic Strain Waves. Science.

[37] Guo J., Bertalan G., Meierhofer D., Klein C., Schreyer S., Steiner B., Wang S., Vieira Da Silva R., Infante-Duarte C., Koch S. (2019). Brain Maturation Is Associated with Increasing Tissue Stiffness and Decreasing Tissue Fluidity. Acta Biomater..

[38] Sack I., Jöhrens K., Würfel J., Braun J. (2013). Structure-Sensitive Elastography: On the Viscoelastic Powerlaw Behavior of in Vivo Human Tissue in Health and Disease. Soft Matter.

[39] Schregel K., Tysiak E., Garteiser P., Gemeinhardt I., Prozorovski T., Aktas O., Merz H., Petersen D., Wuerful J., Sinkus R. (2012). Demyelination Reduces Brain Parenchymal Stiffness Quantified in Vivo by Magnetic Resonance Elastography. Proc. Natl. Acad. Sci. USA.

[40] Streitberger K.-J., Sack I., Krefting D., Pfüller C., Braun J., Paul F., Wuerfel J. (2012). Brain Viscoelasticity Alteration in Chronic-Progressive Multiple Sclerosis. PLoS ONE.

[41] Romano A., Guo J., Prokscha T., Meyer T., Hirsch S., Braun J., Sack I., Scheel M. (2014). In Vivo Waveguide Elastography: Effects of Neurodegeneration in Patients with Amyotrophic Lateral Sclerosis. Magn. Reson. Med..

[42] Murphy M.C., Jones D.T., Jack C.R., Glaser K.J., Senjem M.L., Manduca A., Felmlee J.P., Carter R.E., Ehman R.L., Huston J. (2016). Regional Brain Stiffness Changes across the Alzheimer’s Disease Spectrum. NeuroImage Clin..

[43] Murphy M.C., Huston J., Jack C.R., Glaser K.J., Manduca A., Felmlee J.P., Ehman R.L. (2011). Decreased Brain Stiffness in Alzheimer’s Disease Determined by Magnetic Resonance Elastography. J. Magn. Reson. Imaging.

[44] Coelho A., Sousa N. (2022). Magnetic Resonance Elastography of the Ageing Brain in Normal and Demented Populations: A Systematic Review. Hum. Brain Mapp..

[45] Hiscox L.V., Schwarb H., McGarry M.D.J., Johnson C.L. (2021). Aging Brain Mechanics: Progress and Promise of Magnetic Resonance Elastography. NeuroImage.

[46] Takamura T., Motosugi U., Sasaki Y., Kakegawa T., Sato K., Glaser K.J., Ehman R.L., Onishi H. (2020). Influence of Age on Global and Regional Brain Stiffness in Young and Middle-Aged Adults. J. Magn. Reson. Imaging.

[47] Sack I., Beierbach B., Wuerfel J., Klatt D., Hamhaber U., Papazoglou S., Martus P., Braun J. (2009). The Impact of Aging and Gender on Brain Viscoelasticity. NeuroImage.

[48] Sack I., Streitberger K.-J., Krefting D., Paul F., Braun J. (2011). The Influence of Physiological Aging and Atrophy on Brain Viscoelastic Properties in Humans. PLoS ONE.

[49] Hiscox L.V., Johnson C.L., McGarry M.D.J., Perrins M., Littlejohn A., van Beek E.J.R., Roberts N., Starr J.M. (2018). High-Resolution Magnetic Resonance Elastography Reveals Differences in Subcortical Gray Matter Viscoelasticity between Young and Healthy Older Adults. Neurobiol. Aging.

[50] Johnson C.L., Schwarb H., Horecka K.M., McGarry M.D.J., Hillman C.H., Kramer A.F., Cohen N.J., Barbey A.K. (2018). Double Dissociation of Structure-Function Relationships in Memory and Fluid Intelligence Observed with Magnetic Resonance Elastography. NeuroImage.

[51] Schwarb H., Johnson C.L., McGarry M.D.J., Cohen N.J. (2016). Medial Temporal Lobe Viscoelasticity and Relational Memory Performance. NeuroImage.

[52] Schwarb H., Johnson C.L., Dulas M.R., McGarry M.D.J., Holtrop J.L., Watson P.D., Wang J.X., Voss J.L., Sutton B.P., Cohen N.J. (2019). Structural and Functional MRI Evidence for Distinct Medial Temporal and Prefrontal Roles in Context-Dependent Relational Memory. J. Cogn. Neurosci..

[53] Delgorio P.L., Hiscox L.V., Daugherty A.M., Sanjana F., McIlvain G., Pohlig R.T., McGarry M.D.J., Martens C.R., Schwarb H., Johnson C.L. (2022). Structure-Function Dissociations of Human Hippocampal Subfield Stiffness and Memory Performance. J. Neurosci. Off. J. Soc. Neurosci..

[54] Hiscox L.V., Johnson C.L., McGarry M.D.J., Schwarb H., Van Beek E.J.R., Roberts N., Starr J.M. (2020). Hippocampal Viscoelasticity and Episodic Memory Performance in Healthy Older Adults Examined with Magnetic Resonance Elastography. Brain Imaging Behav..

[55] Pavuluri K., Huston J., Ehman R.L., Manduca A., Jack C.R., Senjem M.L., Vemuri P., Murphy M.C. (2024). Associations between Vascular Health, Brain Stiffness and Global Cognitive Function. Brain Commun..

[56] Destrebecqz A., Peigneux P., Laureys S., Degueldre C., Del Fiore G., Aerts J., Luxen A., Van Der Linden M., Cleeremans A., Maquet P. (2005). The Neural Correlates of Implicit and Explicit Sequence Learning: Interacting Networks Revealed by the Process Dissociation Procedure. Learn. Mem. Cold Spring Harb. Lab. Press.

[57] Rauch S.L., Whalen P.J., Savage C.R., Curran T., Kendrick A., Brown H.D., Bush G., Breiter H.C., Rosen B.R. (1997). Striatal Recruitment during an Implicit Sequence Learning Task as Measured by Functional Magnetic Resonance Imaging. Hum. Brain Mapp..

[58] Hazeltine E. (1997). Attention and Stimulus Characteristics Determine the Locus of Motor—Sequence Encoding. A PET Study. Brain.

[59] Schendan H.E., Searl M.M., Melrose R.J., Stern C.E. (2003). An fMRI Study of the Role of the Medial Temporal Lobe in Implicit and Explicit Sequence Learning. Neuron.

[60] Nasreddine Z.S., Phillips N.A., Bédirian V., Charbonneau S., Whitehead V., Collin I., Cummings J.L., Chertkow H. (2005). The Montreal Cognitive Assessment, MoCA: A Brief Screening Tool for Mild Cognitive Impairment. J. Am. Geriatr. Soc..

[61] Delis D.C., Kramer J.H., Kaplan E., Ober B.A. (2000). California Verbal Learning Test.

[62] Wechsler D. (2008). Wechsler Adult Intelligence Scale.

[63] Schneider W., Eschman A., Zuccolotto A. (2002). E-Prime.

[64] Reed J., Johnson P. (1994). Assessing Implicit Learning with Indirect Tests: Determining What Is Learned about Sequence Structure. J. Exp. Psychol. Learn. Mem. Cogn..

[65] Destrebecqz A., Cleeremans A. (2001). Can Sequence Learning Be Implicit? New Evidence with the Process Dissociation Procedure. Psychon. Bull. Rev..

[66] Fischl B., Salat D.H., Busa E., Albert M., Dieterich M., Haselgrove C., van der Kouwe A., Killiany R., Kennedy D., Klaveness S. (2002). Whole Brain Segmentation: Automated Labeling of Neuroanatomical Structures in the Human Brain. Neuron.

[67] Buckner R.L. (2004). Memory and Executive Function in Aging and AD: Multiple Factors That Cause Decline and Reserve Factors That Compensate. Neuron.

[68] McIlvain G., Cerjanic A.M., Christodoulou A.G., McGarry M.D.J., Johnson C.L. (2022). OSCILLATE: A Low-Rank Approach for Accelerated Magnetic Resonance Elastography. Magn. Reson. Med..

[69] Johnson C.L., Holtrop J.L., Anderson A.T., Sutton B.P. Brain MR Elastography with Multiband Excitation and Nonlinear Motion-Induced Phase Error Correction. Proceedings of the 24th Annual Meeting of the International Society for Magnetic Resonance in Medicine.

[70] Johnson C.L., Holtrop J.L., McGarry M.D.J., Weaver J.B., Paulsen K.D., Georgiadis J.G., Sutton B.P. (2014). 3D Multislab, Multishot Acquisition for Fast, Whole-Brain MR Elastography with High Signal-to-Noise Efficiency. Magn. Reson. Med..

[71] McGarry M.D.J., Van Houten E.E.W., Johnson C.L., Georgiadis J.G., Sutton B.P., Weaver J.B., Paulsen K.D. (2012). Multiresolution MR Elastography Using Nonlinear Inversion. Med. Phys..

[72] McGarry M.D.J., Johnson C.L., Sutton B.P., Van Houten E.E., Georgiadis J.G., Weaver J.B., Paulsen K.D. (2013). Including Spatial Information in Nonlinear Inversion MR Elastography Using Soft Prior Regularization. IEEE Trans. Med. Imaging.

[73] Jenkinson M., Bannister P., Brady M., Smith S. (2002). Improved Optimization for the Robust and Accurate Linear Registration and Motion Correction of Brain Images. NeuroImage.

[74] Jenkinson M., Beckmann C.F., Behrens T.E.J., Woolrich M.W., Smith S.M. (2012). FSL. NeuroImage.

[75] Johnson C.L., Schwarb H., McGarry M.D.J., Anderson A.T., Huesmann G.R., Sutton B.P., Cohen N.J. (2016). Viscoelasticity of Subcortical Gray Matter Structures. Hum. Brain Mapp..

[76] Arani A., Murphy M.C., Glaser K.J., Manduca A., Lake D.S., Kruse S.A., Jack C.R., Ehman R.L., Huston J. (2015). Measuring the Effects of Aging and Sex on Regional Brain Stiffness with MR Elastography in Healthy Older Adults. NeuroImage.

[77] Delgorio P.L., Hiscox L.V., Daugherty A.M., Sanjana F., Pohlig R.T., Ellison J.M., Martens C.R., Schwarb H., McGarry M.D.J., Johnson C.L. (2021). Effect of Aging on the Viscoelastic Properties of Hippocampal Subfields Assessed with High-Resolution MR Elastography. Cereb. Cortex.

[78] Bergs J., Morr A.S., Silva R.V., Infante-Duarte C., Sack I. (2024). The Networking Brain: How Extracellular Matrix, Cellular Networks, and Vasculature Shape the In Vivo Mechanical Properties of the Brain. Adv. Sci..

[79] Freimann F.B., Müller S., Streitberger K.-J., Guo J., Rot S., Ghori A., Vajkoczy P., Reiter R., Sack I., Braun J. (2013). MR Elastography in a Murine Stroke Model Reveals Correlation of Macroscopic Viscoelastic Properties of the Brain with Neuronal Density. NMR Biomed..

[80] Stern Y., Arenaza-Urquijo E.M., Bartrés-Faz D., Belleville S., Cantilon M., Chetelat G., Ewers M., Franzmeier N., Kempermann G., Kremen W.S. (2020). Whitepaper: Defining and Investigating Cognitive Reserve, Brain Reserve, and Brain Maintenance. Alzheimers Dement. J. Alzheimers Assoc..

[81] Hoenig M.C., Dzialas V., Banwinkler M., Asendorf A., Drzezga A., van Eimeren T. (2023). Educational Level and Its Association with Dopamine Transporter Loss in Patients with Parkinson’s Disease. Park. Relat. Disord..

[82] Grafton S.T., Hazeltine E., Ivry R. (1995). Functional Mapping of Sequence Learning in Normal Humans. J. Cogn. Neurosci..

[83] Schwarb H., Johnson C.L., Daugherty A.M., Hillman C.H., Kramer A.F., Cohen N.J., Barbey A.K. (2017). Aerobic Fitness, Hippocampal Viscoelasticity, and Relational Memory Performance. NeuroImage.

[84] Hiscox L.V., McGarry M.D.J., Johnson C.L. (2022). Evaluation of Cerebral Cortex Viscoelastic Property Estimation with Nonlinear Inversion Magnetic Resonance Elastography. Phys. Med. Biol..

[85] Twohy K.E., Kramer M.K., Diano A.M., Bailey O.M., Delgorio P.L., McIlvain G., McGarry M.D.J., Martens C.R., Schwarb H., Hiscox L.V. (2025). Mechanical Properties of the Cortex in Older Adults and Relationships With Personality Traits. Hum. Brain Mapp..

